# RF instrumentation for same‐breath triple nuclear lung MR imaging of ^1^H and hyperpolarized ^3^He and ^129^Xe at 1.5T

**DOI:** 10.1002/mrm.25680

**Published:** 2015-05-13

**Authors:** Madhwesha Rao, Jim M. Wild

**Affiliations:** ^1^ Academic Unit of RadiologyUniversity of SheffieldSheffieldUnited Kingdom

**Keywords:** dual‐tuned RF coil, triple nuclear imaging, pulmonary, lungs, hyperpolarized gas

## Abstract

**Purpose:**

The hyperpolarized gases ^3^He and ^129^Xe have distinct properties and provide unique and complementary functional information from the lungs. A triple‐nuclear, same‐breath imaging examination of the lungs with ^1^H, ^3^He, and ^129^Xe can therefore provide exclusive functional information from the gas images. In addition, the ^1^H images provide complementary co‐registered structural information in the same physiological time frame. The goal of this study was to design an RF system for triple nuclear lung MRI at 1.5T, consisting of a dual‐tuned transceiver coil for ^3^He and ^129^Xe, RF switches and a nested ^1^H receiver array.

**Methods:**

A dual‐tuned transmit‐receive dual‐Helmholtz RF coil for ^3^He and ^129^Xe was designed and constructed to work in unison with a nested ^1^H receiver array.

**Results:**

Triple‐nuclear imaging (structural and ventilation) and apparent diffusion coefficient mapping of the human lungs was performed in the same breath‐hold using the integrated RF system. B_1_ maps and volumetric ventilation imaging using a three‐dimensional, balanced steady‐state free precession pulse sequence performed with both hyperpolarized ^3^He and ^129^Xe indicate good stand‐alone performance of the coil for the respective nucleus.

**Conclusion:**

Triple‐nuclear same‐breath lung imaging with a dual‐tuned coil (^3^He and ^129^Xe) and a nested ^1^H array has been demonstrated with a custom RF system. **Magn Reson Med 75:1841–1848, 2016. © © The Authors Magnetic Resonance in Medicine published by Wiley Periodicals, Inc. on behalf of International Society for Magnetic Resonance in Medicine**.

## INTRODUCTION

Imaging the lungs with inhaled hyperpolarized gases ^3^He and ^129^Xe has been shown to provide functional information that cannot be accessed with proton (^1^H) MRI or other imaging modalities 1, 2, 3, 4, 5, 6. The two gases have distinct physical properties, which provide different but complementary functional information 7, 8, 9. The ability to image both nuclei in the same breath alongside the ^1^H anatomical images adds further structural and functional sensitivity to the acquisition. ^3^He is highly diffusive when compared with ^129^Xe 10, 11, 12, and the visualization and quantification of lung ventilation and diffusion with these two gases at the same time can help address important physiological questions such as the position of the diffusion–convection front in the lungs. The capability to measure the diffusivity of both ^3^He and ^129^Xe gases in the same lung inflation level also provides added information for measuring and modeling lung microstructure based on their measured apparent diffusion coefficients (ADC) 13. ^129^Xe is also denser and more viscous than ^3^He and as such has different fluid dynamic properties that define airflow in the airways, which can be measured with phase contrast MRI 14. ^129^Xe has the added feature that it is soluble in blood and has a wide range of chemical shift, which enables quantification of perfusion and gas exchange in the lungs 8, 9, 15, 16, 17. In recent years, MRI of perfluorinated ^19^F gases has also gained interest 18, 19, 20 as another MR‐sensitive gaseous tracer of regional lung function.

Therefore, same‐breath, multinuclear lung imaging with ^3^He‐^129^Xe mixtures and ^1^H MRI provides a unique combination of functional and structural information that is spatio‐temporally coregistered in the same physiological time frame 21, 22. Preliminary studies have used separate and spatially nested transmit‐receive (T‐R) coils for each nucleus 22. The reliance on the ^1^H MR system's birdcage body coil for signal reception constrains the signal‐to‐noise ratio (SNR) in ^1^H images of the lung, which is already limited by the low proton density of lung parenchyma. In a recent study 23, we showed that the ^1^H lung SNR in same‐breath imaging can be improved with a nested ^1^H receive array, which is compatible with operation with either a ^3^He or a ^129^Xe T‐R coil.

The motivation of this study was the design and construction of an integrated radiofrequency (RF) coil and T‐R switching system for triple nuclear lung imaging in the same breath. To achieve this, we developed a new dual‐tuned flexible T‐R RF coil to operate in quadrature for both ^3^He and ^129^Xe at 1.5T. For ^1^H imaging, we incorporated the ^1^H array developed in our previous study designed to nest within either ^3^He or ^129^Xe T‐R RF coils 23. With the developed RF instrumentation, we demonstrated triple nuclear same‐breath lung imaging with hyperpolarized ^3^He and ^129^Xe ventilation images and ^1^H anatomical images. With the same system ADC measurement of mixtures of ^3^He and ^129^Xe were performed in the same breath at a particular lung inflation state.

## METHODS

### ^3^He and ^129^Xe Dual‐Tuned Coil Design

**Figure 1:**
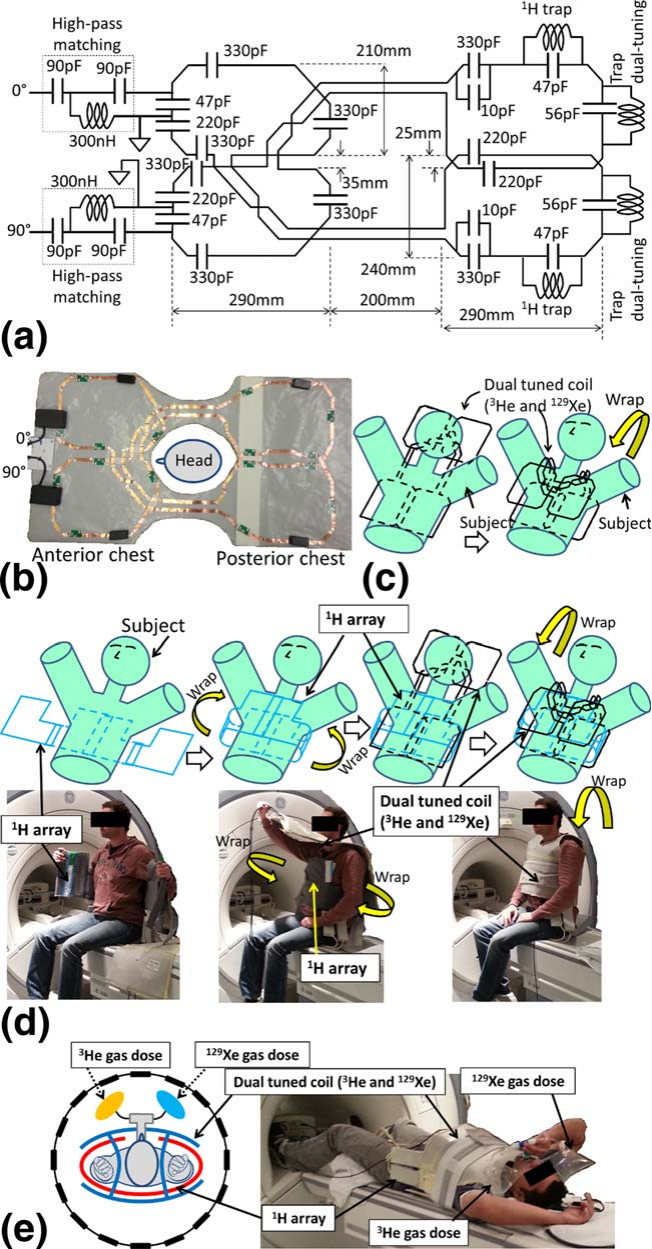
**a:** Schematic of the dual‐tuned flexible T‐R coil for ^129^Xe and ^3^He. The in‐phase and quadrature‐phase ports are marked with 0° and 90°, respectively. **b:** Picture of the dual‐tuned flexible T‐R coil for ^129^Xe and ^3^He. The in‐phase and quadrature‐phase ports are marked with 0° and 90°, respectively. The plastic housing at the port (0°, 90°) consists of high pass matching circuits (90 pF, 90 pF, and 300 nH) marked in the schematic and the two loop capacitors (47 pF and 220 pF). **c:** Illustration of application of dual‐tuned T‐R coil on the subject for same‐breath ADC measurement. **d:** Illustration of ^1^H array and dual‐tuned coil nested for triple nuclear imaging. **e:** Picture of the setup on the scanner. The picture indicates the mouthpiece and the two Tedlar bags affixed to it.

A dual‐tuned (^3^He‐^129^Xe) flexible quadrature T‐R coil was constructed in‐house. The conducting elements were made from self‐adhesive copper tape (FE‐5100–5276‐7; 3M, Bracknell, UK) of 66‐μm thickness and 6‐mm width, which was fixed on a substrate of 0.5‐mm‐thick polytetrafluoroethylene (Direct Plastics, Sheffield, UK) as shown in Figure 1b. The capacitors used on the resonant circuit were of 10C package (Dalian Dalicap Technology Co., Ltd, Dalian, China). The thickness of the array with the foam was 6 mm (3 mm each side). The dual‐tuned flexible T‐R coil was a dual Helmholtz‐like pair of quadrature design in which the Helmholtz for the in‐phase resonance of the quadrature spans the anterior right lung to posterior left lung, connected over left trapezius. Similarly, the Helmholtz for the quadrature‐phase resonance spans the anterior left lung to posterior right lung, via the right trapezius. The cross‐over of the copper strip for each of the Helmholtz pairs (which forms a “figure eight” topology) was positioned such that it was within the other resonant element (anterior) and was balanced on either side to minimize coupling as shown in Figure 1a and 1b. The schematic of the dual‐tuned T‐R coil circuit is shown in Figure 1a, and a photograph is shown in Figure 1b. The assembled topology of the flexible coil constitutes a bib design wrapped around the subject longitudinally, as shown in Figure 1c. Both the elements of the dual Helmholtz were fitted with two traps; one trap at the ^1^H frequency to enable ^1^H imaging with this coil in situ and the other trap to dual‐tune the coil to the ^129^Xe and ^3^He Larmor frequencies. The trap design was based on the formalism established in our earlier study for multituned resonators 23, the frequency of the trap for dual‐tuning was 47.81 MHz. A high‐pass matching circuit was used to match the coil at both resonant frequencies of ^3^He (48.62 MHz) and ^129^Xe (17.65 MHz) at 1.5T. The ^1^H trap was tuned with a 47‐pF capacitor and a seven‐turn wire wound inductor with a diameter of 6 mm. The trap for dual‐tuning the coil was tuned with a 56‐pF capacitor and a nine‐turn wire wound inductor with a diameter of 6 mm. Wire wound inductors were constructed from 21 AWG insulated copper wire. RF measurements were performed with an Agilent 5061B Network Analyzer (Keysight Technologies, Santa Rosa, California, USA). For the RF measurement, the dual‐tuned coil was wrapped longitudinally around the thorax of the subject, as shown in Figure 1c. This coil was designed to work with full functionality when the four‐channel ^1^H chest receiver array from our earlier study 23 was nested inside for in situ high SNR ^1^H lung imaging.

### MR Imaging Methods for ^3^He, ^129^Xe and ^1^H

All in vivo imaging with ^3^He and ^129^Xe was performed with approval from the National Research Ethics Committee. The imaging was performed on a healthy male volunteer (age, 31 years; height, 185 cm; weight, 89 kg). Lung MRI was performed on a GE whole body 1.5T Signa HDx system with ^3^He and ^129^Xe gas polarized with spin exchange optical pumping 24. The gas dosage and the imaging and pulse sequence parameters used for all three nuclei are shown in Table 1. The hyperpolarized ^3^He and ^129^Xe gas was delivered in separate Tedlar bags and was mixed at the mouth piece at the time of inhalation, as illustrated in Figure 1e. ^3^He had polarization of 25% (≈100% of He is ^3^He). ^129^Xe had a polarization of 40%–50% (87% of Xe is ^129^Xe).

**Table 1:** Gas Mixture Dosage, Imaging Parameters, and Pulse Sequence Parameters Used in the Study

Measurement	Lung structure and ventilation	ADC	Whole lung ventilation	Flip angle map
Physiological details	Triple‐nuclear, same breath	Dual‐nuclear, same breath	Single‐nuclear, separate breath	Single‐nuclear, separate breath
RF coil	^1^H array, dual‐tuned coil	Dual‐tuned coil	Dual‐tuned coil	Dual‐tuned coil
Nuclei	^1^H, ^3^He, ^129^Xe	^3^He, ^129^Xe	^3^He, ^129^Xe	^3^He, ^129^Xe
Dosage (mL)				
^ 1^H	—	—	—	—
^ 3^He	350	300	200	50
^ 129^Xe	500	500	500	100
Flip angle				
^ 1^H	50°	—	—	—
^ 3^He	8°	9°	10°	—
^ 129^Xe	9°	10°	10°	—
TE (ms)				
^ 1^H	0.9	—	—	—
^ 3^He	1.1	4.8	0.6	1.1
^ 129^Xe	3.6	12.5	2.1	3.6
TR (ms)				
^ 1^H	2.9	—	—	—
^ 3^He	3.6	10	1.9	3.6
^ 129^Xe	18.9	27	6.4	18.9
Matrix				
^1^H				
Phase	192	—	—	—
Frequency	256	—	—	—
^3^He				
Phase	104	48	82	52
Frequency	80	64	80	44
^129^Xe				
Phase	78	48	82	52
Frequency	64	64	80	44
Slice thickness (mm)				
^ 1^H	15	—	—	—
^ 3^He	15	15	4	200
^ 129^Xe	15	15	10	200
Number of slices				
^ 1^H	3			
^ 3^He	3	2	46	1
^ 129^Xe	3	2	24	1
Field of view (cm)				
^ 1^H	40			
^ 3^He	40	44	40	40
^ 129^Xe	40	44	40	40
Axis	2D, coronal	2D, coronal	Coronal	2D, coronal
Pulse sequence	bSSFP, FSGRE	FSGRE	3D bSSFP	FSGRE
Imaging time (s)				
^ 1^H	1	—	—	—
^ 3^He	2	6	7	0.9
^ 129^Xe	4	8	13	2.4
Multiphase				
^ 1^H	—	—	—	—
^ 3^He	—	—	—	6
^ 129^Xe	—	—	—	6
b Value (s · cm^−2^)				
^ 1^H	—	—	—	—
^ 3^He	—	1.6	—	—
^ 129^Xe	—	8	—	—
Corresponding figure				
^ 1^H	3a, 3d, 3e	—	—	—
^ 3^He	3b, 3d	2e	4a	2c
^ 129^Xe	3c, 3e	2f	4b	2d

Abbreviations: ADC, apparent diffusion coefficient; bSSFP, balanced steady‐state free precession; FSGRE, fast spoiled gradient echo.

### RF Signal Routing and Calibration

To route the transmit RF signal (^3^He‐^129^Xe) from the appropriate T‐R switch on the scanner to the dual‐tuned coil and to route the received RF signal (^3^He‐^129^Xe) from the dual‐tuned coil back to the appropriate T‐R switch on the scanner, a 2‐kW rated coaxial antenna RF switch (CX‐SW2PL; Watson, Essex, UK) was used. The RF power required for the desired flip angle for the ^3^He and ^129^Xe sequences was calculated based on a standard calibration procedure, whereby the rate of depletion of polarization was calculated from the decay of signal resulting from a set of hard RF pulse‐acquires of equal amplitude. The period to prescribe calibration values on the spectrometer between the end of imaging a particular nucleus and initiation of the sequence for imaging the next nucleus was less than 4 s. The time required to operate the RF switch manually between acquisitions was 3 s.

### Same‐Breath ADC (^3^He and ^129^Xe) and Triple Nuclear (^3^He, ^129^Xe, and ^1^H) Structure and Ventilation Lung Imaging Methods

For same‐breath ADC measurement, the dual‐tuned ^3^He‐^129^Xe coil was wrapped longitudinally as shown in Figure 1c, without the ^1^H array nested inside. To demonstrate same‐breath ADC maps, two sets of ADC measurements were acquired back‐to‐back in a single breath, with ^3^He ADC measurement followed by ^129^Xe measurement. The imaging parameters are shown in Table 1.

For triple‐nuclear lung imaging, the dual‐tuned ^3^He ‐^129^Xe coil and the ^1^H array from our earlier study 23 were nested as shown in Figure 1d. To demonstrate imaging of all three nuclei in the same breath, three sets of images were acquired back‐to‐back in a single breath in the order, with ^3^He imaging followed by ^129^Xe imaging, in turn followed by ^1^H imaging. The imaging parameters are shown in Table 1.

The T_1_ of hyperpolarized gases when inhaled into the lungs is sensitive to the oxygen partial pressure in the lung during the breath‐hold 25. ^3^He is more sensitive to this effect because the gyromagnetic ratio of ^3^He is approximately three times larger than that of ^129^Xe, as such the dipolar coupling to the electrons in the paramagnetic oxygen molecule is stronger. This rationale for the order of acquisition is ^3^He followed by ^129^Xe, in turn followed by ^1^H.

### Flip Angle Mapping and High‐Resolution Imaging Performance of the Coil as a Stand‐Alone T‐R Coil for ^3^He and ^129^Xe

Flip angle maps of the dual‐tuned coil at the ^3^He and ^129^Xe frequencies were calculated by measuring the depletion of polarization of the hyperpolarized gas ^3^He and ^129^Xe at each voxel in the lungs by repeated imaging at breath‐hold with a two‐dimensional spoiled gradient echo sequence. The imaging parameters for this measurement are shown in Table 1, and T_1_ relaxation was neglected when calculating the flip angle. In addition, to demonstrate the coil's performance as a stand‐alone ^3^He or ^129^Xe T‐R coil (without the ^1^H array in situ), high‐resolution, three‐dimensional (3D) imaging data sets were acquired with a 3D balanced steady state sequence 26 with imaging parameters as shown in Table 1.

## RESULTS

### Dual‐Tuned Coil RF Performance

**Figure 2:**
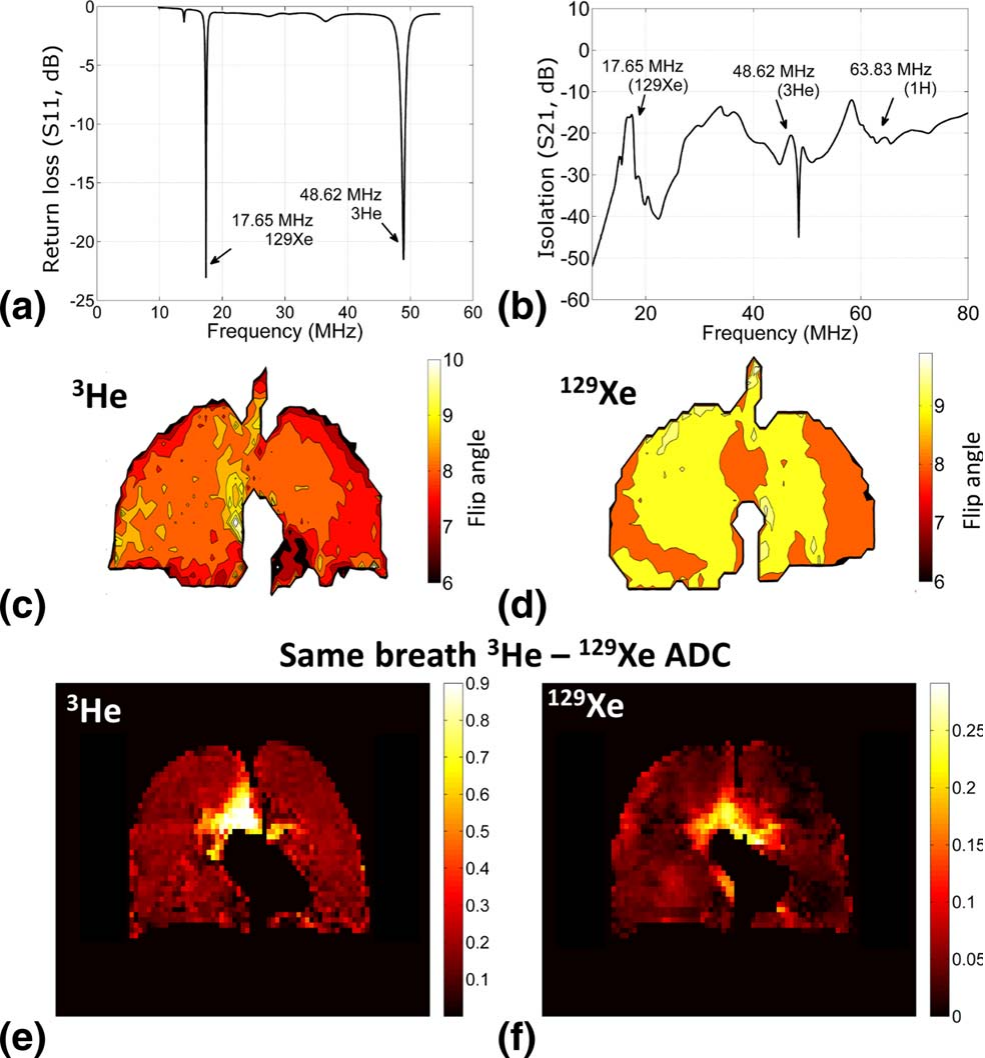
**a:** Matching of dual‐tuned coil at 17.65 MHz and 48.62 MHz. **b:** Isolation between dual‐tuned coil and ^1^H array over frequency span of 10–80 MHz. **c:** Flip angle map of dual‐tuned coil for ^3^He. **d:** Flip angle map of dual‐tuned coil for ^129^Xe. The color bars indicate the flip angle in degrees. e**, f:** ADC measurement was taken in the same breath for ^3^He (e) and ^129^Xe (f). The color bars indicate the ADC in cm^2^ · s^−1^.

The two traps on the coil at 47.81 MHz and 63.86 MHz (^1^H trap) generated three resonant modes at 17.65 MHz (^129^Xe Larmor frequency), 48.62 MHz (^3^He Larmor frequency), and 79.2 MHz. The isolation between the two ports of the Helmholtz was less than −15 dB. The quality (Q) factor of the dual‐tuned coil at the ^129^Xe Larmor frequency (17.65 MHz) was 61 in the unloaded condition and 17 in the loaded condition. The Q factor of the dual‐tuned coil at the ^3^He Larmor frequency (48.62 MHz) was 32 in the unloaded condition and 7 in the loaded condition. Thus, the ratio of Q factor unloaded to loaded condition was 3.5 at the ^129^Xe Larmor frequency (17.65 MHz) and 4.5 at the ^3^He Larmor frequency (48.62 MHz). Under the loaded condition, the dual‐tuned coil was matched to less than −20 dB at both ports at both the ^129^Xe (17.65 MHz) and ^3^He (48.62 MHz) Larmor frequencies, as shown in Figure 2a. The isolation between the dual‐tuned coil and the ^1^H array was less than −15 dB, as shown in Figure 2b. Flip angle maps from the dual‐tuned coil for the transmit RF power prescribed for the nominal flip angles used for triple nuclear same‐breath imaging and ADC measurement (Table 1) are shown in Figure 2c for ^3^He and Figure 2d for ^129^Xe. The standard deviation of the flip angle map was calculated to be 0.7° (mean = 8°) for ^3^He and 0.3° (mean = 9°) for ^129^Xe.

### Multinuclear Lung Imaging

Same‐breath ADC measurement of ^3^He and ^129^Xe performed in the same lung‐inflation state is shown in Figure 2e and 2f. The ^3^He ADC map shown in Figure 2e and the ^129^Xe ADC map shown in Figure 2f were acquired in the same breath.

**Figure 3:**
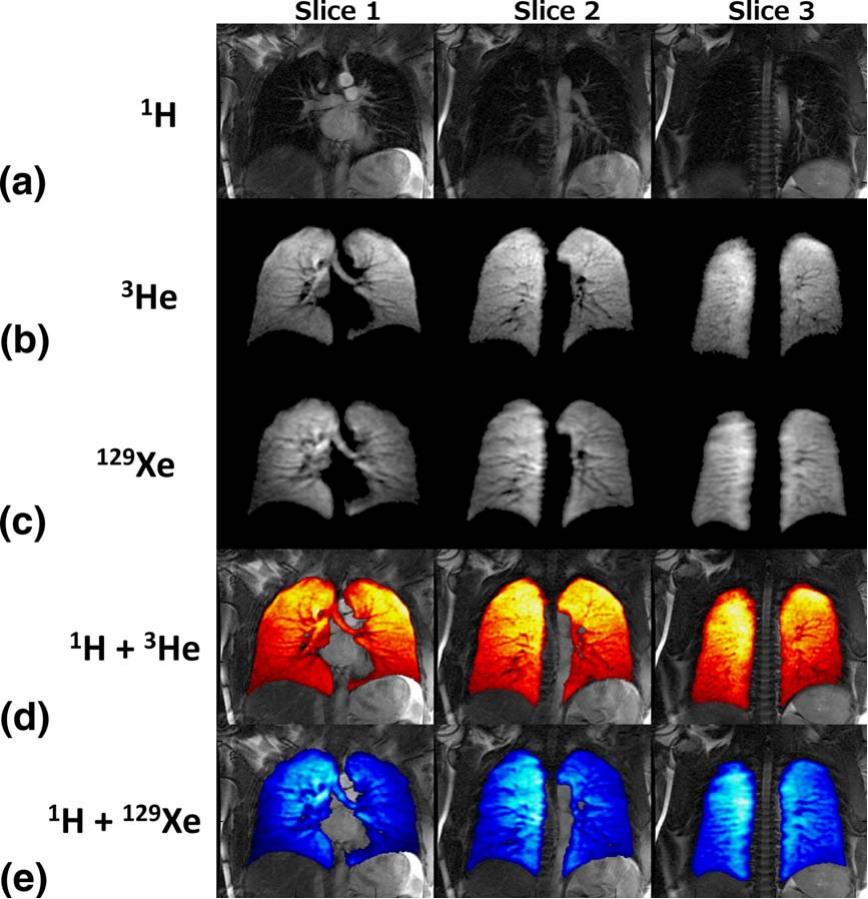
**a:** ^1^H images from lungs. **b:** Same‐breath ^3^He images from lungs. **c:** Same‐breath ^129^Xe images from lungs. **d:** ^3^He images superimposed over ^1^H images. **e:** ^129^Xe images superimposed over ^1^H images.

Same‐breath triple nuclear lung (structure and ventilation) images are shown in Figure 3. The ^1^H images shown in Figure 3a, ^3^He images shown in Figure 3b, and ^129^Xe images shown in Figure 3c, all of which were acquired in the same breath, are coregistered as shown in superimposed images in Figure 3d and 3e.

**Figure 4:**
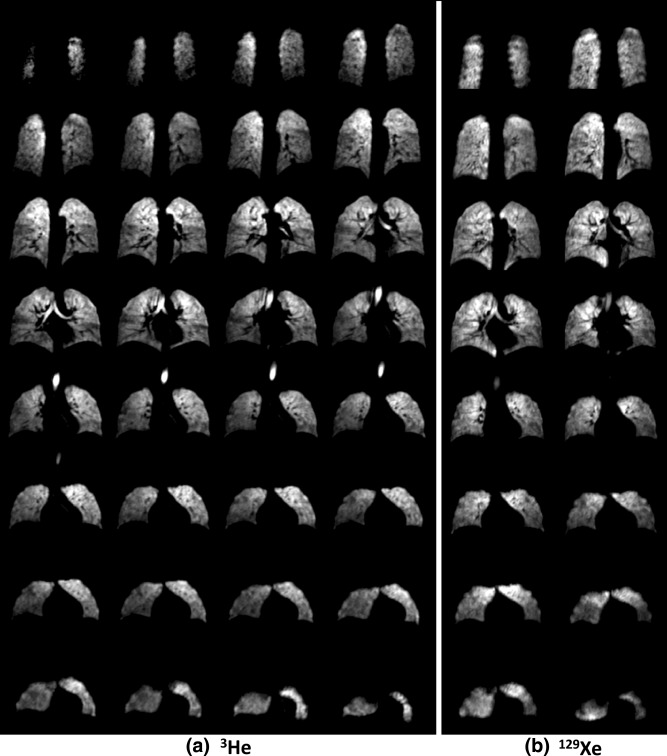
Hyperpolarized gas images of the lungs from 3D balanced steady‐state free precession sequence with dual‐tuned coil in a stand‐alone configuration (without ^1^H array nested). **a:** Hyperpolarized ^3^He gas. **b:** Hyperpolarized ^129^Xe gas.

Volumetric ventilation images from the 3D balanced steady‐state free precession sequence for the coil in operation as a stand‐alone transceiver for ^3^He and ^129^Xe are shown in Figure 4a and Figure 4b, respectively.

## DISCUSSION

The construction of the flexible dual‐tuned coil is in the form of a bib, which enables a close fit to the subject's thorax irrespective of body type. The design was optimized to the typical subject size mentioned earlier. As the shape/form deviates from the optimal design with other body types, the distributed inductance and T‐R efficiency of the dual‐tuned coil changes accordingly. The B_1_ field homogeneity of the dual Helmholtz design is inherently inferior to that of a birdcage design 27, and the flexibility of the dual‐tuned coil adds some variability in this respect. Considering the typical anatomy of a torso, the distance between the RF coil and lung air spaces generally increases from superior (upper) to inferior (lower). This means that sensitivity in the lower lung is reduced for two reasons: first, due to proximity of the conducting elements to the lungs, and second, as the parallel condition for a Helmholtz pair is disrupted. Despite these factors, the observed B_1_ transmit homogeneity (variation in flip angle, 3% for ^129^Xe and 9% for ^3^He) is comparable to 28 or better than 29 studies reported previously using single‐tuned flexible T‐R coils for ^3^He and ^129^Xe lung imaging.

Because the RF switches are currently manually operated, and the spectrometer has an inherent delay time for precalibration for each nucleus, the method is not currently compatible with repetition time resonant frequency interleaved imaging, as demonstrated in our earlier study with same‐breath ^3^He‐^1^H lung imaging 22. It should be noted that this limitation is not due to the RF coil design or configuration; instead, it is due to the MR system, which supports only one spectrometer T‐R switch (single‐nucleus) to be actively connected at any given point in time (in addition to ^1^H). Both the dual‐tuned RF coil and the nested ^1^H array from the earlier study 23 are capable of operating simultaneously. If we consider the coil's operation as part of the system for triple nuclear imaging, 50%–60% of the time (ie, 18–20 s of the breath‐hold) is consumed by switching the spectrometer between the nuclei. This can be reduced with the appropriate spectrometer software engineering and using electrically driven RF switches (eg, PIN diodes and Field Effect Transistor).

The free diffusion (in air) of ^3^He is 0.88 cm^2^ · s^−1^ 30, 31; in this study, we report 0.85 cm^2^ · s^−1^ for ^3^He in the trachea (slightly lower than ^3^He free diffusion). The free diffusion of ^129^Xe is 0.14 cm^2^ · s^−1^ 31; in this study, we report 0.22 cm^2^ · s^−1^ for ^129^Xe in the trachea. The higher ADC value for ^129^Xe in the trachea, we presume is due to its mixture with the highly diffusive ^3^He (as shown in Table 1). In the ventilation images, any observed asymmetry beyond what can be attributed to the measured variation/asymmetry in the flip angle was verified to be caused by the distribution of ^3^He and ^129^Xe as a gas mixture in the lung (variation in the local concentration of the gases). These findings are currently being investigated in future work studying the physiology of gas mixing in the lung with the two gases.

In contrast to our previous triple nuclear same‐breath lung imaging experiments demonstrated at 3T on a Philips system using the ^1^H body T‐R coil, a ^3^He birdcage T‐R coil, and a nested ^129^Xe T‐R vest coil 22, the design used in this study at 1.5T has several potential benefits. First, from the coil perspective, the use of the dual‐tuned ^3^He‐^129^Xe coil minimizes the number of individually tuned coils, and the nested ^1^H array 23 improves the ^1^H SNR by closer proximity to the lung. Applications of this triple nuclear RF system for lung MRI are manifold and allow the different physical and physiological properties of the two gases to be explored in the same time course with added provision of high‐quality and coregistered ^1^H structural images.

In conclusion, we have demonstrated a system for triple nuclear same‐breath lung imaging of ^1^H with hyperpolarized gases ^3^He and ^129^Xe at 1.5T using a custom integrated RF system. This system incorporates a new design of dual‐tuned RF coil for ^3^He and ^129^Xe and RF switches, together with a nested receiver array for ^1^H imaging. With this system, we have demonstrated high‐quality, same‐breath ^1^H with ^3^He and ^129^Xe ventilation imaging and the capability for ADC mapping of ^3^He and ^129^Xe in the same lung‐inflation state. In addition, the image quality on all three nuclei is comparable with those acquired with separate RF coils for the given nucleus.
